# Selection, hybridization, and the evolution of morphology in the Lake Malaŵi endemic cichlids of the genus *Labeotropheus*

**DOI:** 10.1038/s41598-018-34135-x

**Published:** 2018-10-26

**Authors:** Michael J. Pauers, Kelsey R. Fox, Robert A. Hall, Kesha Patel

**Affiliations:** 1 Section of Vertebrate Zoology, Milwaukee Public Museum, 800 W. Wells Street, Milwaukee, Wisconsin 53233 USA; 2 Department of Biological Sciences, University of Wisconsin-Milwaukee at Waukesha, 1500 N. University Drive, Waukesha, Wisconsin 53188 USA; 3 School of Freshwater Sciences, University of Wisconsin-Milwaukee, 600 E. Greenfield Avenue, Milwaukee, Wisconsin 53204 USA; 4 University of Wisconsin-Madison, Madison, WI 53708 USA

## Abstract

The cichlid fishes of Lake Malaŵi are the paramount example of adaptive radiation in vertebrates. Evidence of their astounding diversity is perhaps most visible in their adaptations for obtaining food; the genus *Labeotropheus*, due to their prominent snouts, are an interesting example of an extreme adaptation for feeding. Two different body types are found in this genus: a deep-bodied form (e.g., *L*. *fuelleborni*) found most often in turbulent shallow water; and a slender bodied form (e.g., *L*. *trewavasae*) found in structurally-complex deep water habitats. Here we test the hypothesis that *L*. *trewavasae* should suffer a loss in fitness, measured as growth rate, if raised in turbulence; additionally, we examined growth and morphology of *L*. *fuelleborni* and *L*. *fuelleborni* x *L*. *trewavasae* hybrids under these conditions. We did find the predicted loss of fitness in turbulent-raised *L*. *trewavasae*, but found no loss of fitness for *L*. *fuelleborni* in either condition; hybrids, due to an unusual morphology, performed better in turbulent as opposed to control conditions. Fitness in turbulent conditions was dependent upon morphology, with deeper bodies and upturned neurocrania allowing a greater growth rate under these conditions. Directional selection on morphology was crucial in the evolution of morphology in the *Labeotropheus*.

## Introduction

The cichlid fishes of Lake Malaŵi are one of the most fascinating examples of adaptive radiation and speciation; indeed, no other group of vertebrates can match either their sheer number of species or vast diversity of form and function. Nowhere is this diversity of form and function more prevalent than in the variety of adaptations these fishes have for acquiring food e.g.^1,2^. These fishes have evolved a striking number of ways to divide and subdivide what appear to be homogenous resources, yet the astounding array of craniofacial, oral, dental, and pharyngeal adaptations they have allow the various cichlid species to exploit very specific portions of the available food resources^3–5^, thus promoting the coexistence of so many disparate species.

Amongst the most unique and unusual feeding adaptations displayed by any of the cichlids of Lake Malaŵi is that of the genus *Labeotropheus*. The diagnostic feature shared by all members of this small genus is an enlarged snout of fibrous connective tissue that hangs over an inferior, subterminal mouth, with which all of these species scrape filamentous, epilithic algae^1,6–8^. This snout has been the focus of many hypotheses regarding how the *Labeotropheus* feed, and it is generally assumed to act as a fulcrum with which the fish contacts the substrate, while the jaws work to remove the attached algae; this process is exquisitely described by Fryer^1^; see also^3,8–10^. The uniqueness of this feeding apparatus has made various species of *Labeotropheus* favorite subjects for studying the evolution and ecology of cichlid feeding^8,11–13^.

Long overlooked in the study of *Labeotropheus* feeding is the role body shape may play in the different environments in which members of this genus are found. While Fryer^1^ was the first to note that its characteristic snout allowed species of *Labeotropheus* to feed almost parallel to the substrate, Ribbink *et al*.^3^ further suggested that this could be a significant advantage for *L*. *fuelleborni*, a deep-bodied species that primarily inhabits the shallow, wave-swept areas along the shore of Lake Malaŵi. These authors suggested that by keeping its stocky body parallel to a horizontal rock surface, *L*. *fuelleborni* would be better able to maintain its position in this turbulent habitat^3^. Similarly, Konings^14^ observed that *L*. *trewavasae*, a slender-bodied species found at deeper depths than *L*. *fuelleborni*, seems to use its slim body to penetrate into crevices among rocks, or to feed from the vertical surfaces of rocks, in order to avoid ingesting sediment. To date, neither of these observations has been experimentally confirmed.

If these divergent morphologies are adaptations to their respective habitats, then individuals forced to feed and grow in a mismatched habitat should suffer fitness-related consequences. Specifically, if *L*. *trewavasae* is grown in a turbulent habitat, we would expect that it should experience a reduced growth rate; growth rate being a commonly-used and reasonable proxy for fitness in fishes e.g.^15,16^. Alternatively, it might be possible that such individuals adapt to turbulence by adopting a morphology similar to that of *L*. *fuelleborni*; i.e., a deep, stocky, more massive body. Here, we test these hypotheses and predictions in *L*. *fuelleborni*, *L*. *trewavasae*, and their hybrids.

## Results

### Dimensionality of morphology

**Figure 1:**
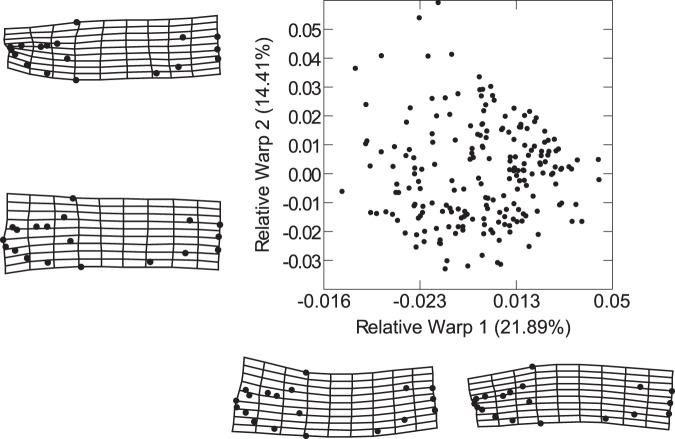
The overall dimensionality of morphology among all three species. RW 1 explains 21.89% of the observed variation in body shape, and primarily accounts for the difference between a slender body with a downwardly-pointing head and a deep body with an upwardly-tilted head. RW 2 explains 14.41% of the variation in body shape, and accounts for the difference between a slender body with a dorsoventrally-compressed head and a deep body with an expanded craniofacial region. The deformation grids show the morphologies associated with the extremes of each axis, and are positioned accordingly.

Relative Warp (RW) 1 explains 21.89% of the observed variation in body shape amongst the experimental fishes. This axis primarily distinguishes between two opposing morphologies: a slender-bodied form with a dorsoventrally compressed, downturned head at the positive end; versus a deeper-bodied form with an expanded, upturned head at the negative. RW 2 accounts for 14.41% of the variation in body shape, and distinguishes between a very slender body with a reduced snout; versus a distinctly deeper body that features a prominent, enlarged snout (Fig. 1). In this preliminary examination of morphology, it appears that the slender *L*. *trewavasae* specimens are clustered within the positive quadrant, while the robust *L*. *fuelleborni* individuals seem to be confined to the negative quadrant.

### Morphological differences between species and treatments

**Figure 2:**
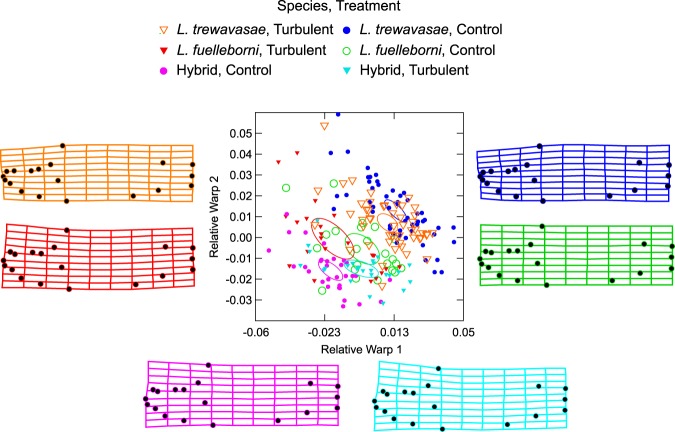
Differences in body shape among treatment groups at Day 45. This is the same RW plot as shown in Fig. 1, but the treatment groups have been identified by symbol (circle = control treatment; triangle = turbulent treatment) and color (green = *L*. *fuelleborni* control; red = *L*. *fuelleborni* turbulent; pink = hybrid control; aqua = hybrid turbulent; blue = *L*. *trewavasae* control; orange = *L*. *trewavasae* turbulent); the colors of the deformation grids also correspond to these treatment groups. In the turbulent-raised *L*. *fuelleborni* and*L*. *trewavasae*, note the expanded and upturned heads, especially in comparison to their respective controls. In the hybrid fishes, note the deep body but exaggerated breadth of the craniofacial area in the control cohort, especially in comparison to the *L*. *fuelleborni* and *L*. *trewavasae* controls; conversely, the turbulent-raised hybrids are much more similar to the turbulent-raised cohorts of the purebred parental species.

By the end of the experiment, while clear differences in body shape were evident among species, there appear to be some morphological commonalities among species between treatments. RW 1 distinguishes the species, as well as the experimental treatments within species (Table 1). The turbulent-reared individuals of both *L*. *fuelleborni* and *L*. *trewavasae* are situated negatively, with respect to the control fish, along RW 1. The hybrid individuals of the control and turbulent treatments are similarly distinguished, although their placements relative to each other are the reverse of the parental species; the turbulent individuals are situated positively relative to the control fish (Fig. 2). RW 2 separates the species from one another, with *L*. *trewavasae* located at the positive end of this axis, the hybrids at the negative end, and *L*. *fuelleborni* situated near the midpoint of RW 2 (Fig. 2; Table 1).

**Table 1:** Differences along each Relative Warp (RW) axis among species and treatments.

Analysis of effects on RW 1 (n = 202; Multiple R^2^ = 0.417)					
Variable	Sum of Squares	df	Mean Square	F	p
Species	0.029	2	0.014	54.714	≤ 0.001
Treatment	0.000	1	0.000	0.249	0.618
Species*Treatment	0.007	2	0.003	12.924	≤ 0.001
Error	0.051	196	0.000		
Bonferroni-corrected pairwise differences in RW 1 among species*treatment (LFC = ***L***. ***fuelleborni*** **Control; LHC = *Labeotropheus*** **hybrid Control;** ***L***. ***trewavasae*** **Control; LFT** = ***L***. ***fuelleborni*** **Turbulent; LHT** = ***Labeotropheus*** **hybrid Turbulent; LTT** = ***L***. ***trewavasae*** **Turbulent)**					
	**LFC**	**LHC**	**LTC**	**LFT**	**LHT**
LHC	−0.017**				
LTC	0.018***	0.034***			
LFT	−0.016*	0.001	−0.033***		
LHT	−0.001	0.016**	−0.018***	0.015*	
LTT	0.013*	0.030***	−0.004	0.029***	0.014**
**Analysis of effects on RW 2 (n** = **202; Multiple R**^**2**^ = **0**.**433)**					
**Variable**	**Sum of Squares**	**df**	**Mean Square**	**F**	**p**
Species	0.023	2	0.012	70.413	≤ 0.001
Treatment	0.000	1	0.000	0.001	0.981
Species*Treatment	0.001	2	0.001	3.030	0.051
Error	0.033	196	0.000		
**Bonferroni-corrected pairwise differences in RW 2 among species**					
		***L***. ***fuelleborni***		***Labeotropheus*** **hybrid**	
*Labeotropheus* hybrid		−0.012***			
*L*. *trewavasae*		0.013***		0.025***	

*p ≤ 0.05; **p ≤ 0.01; ***p ≤ 0.001.

**Figure 3:**
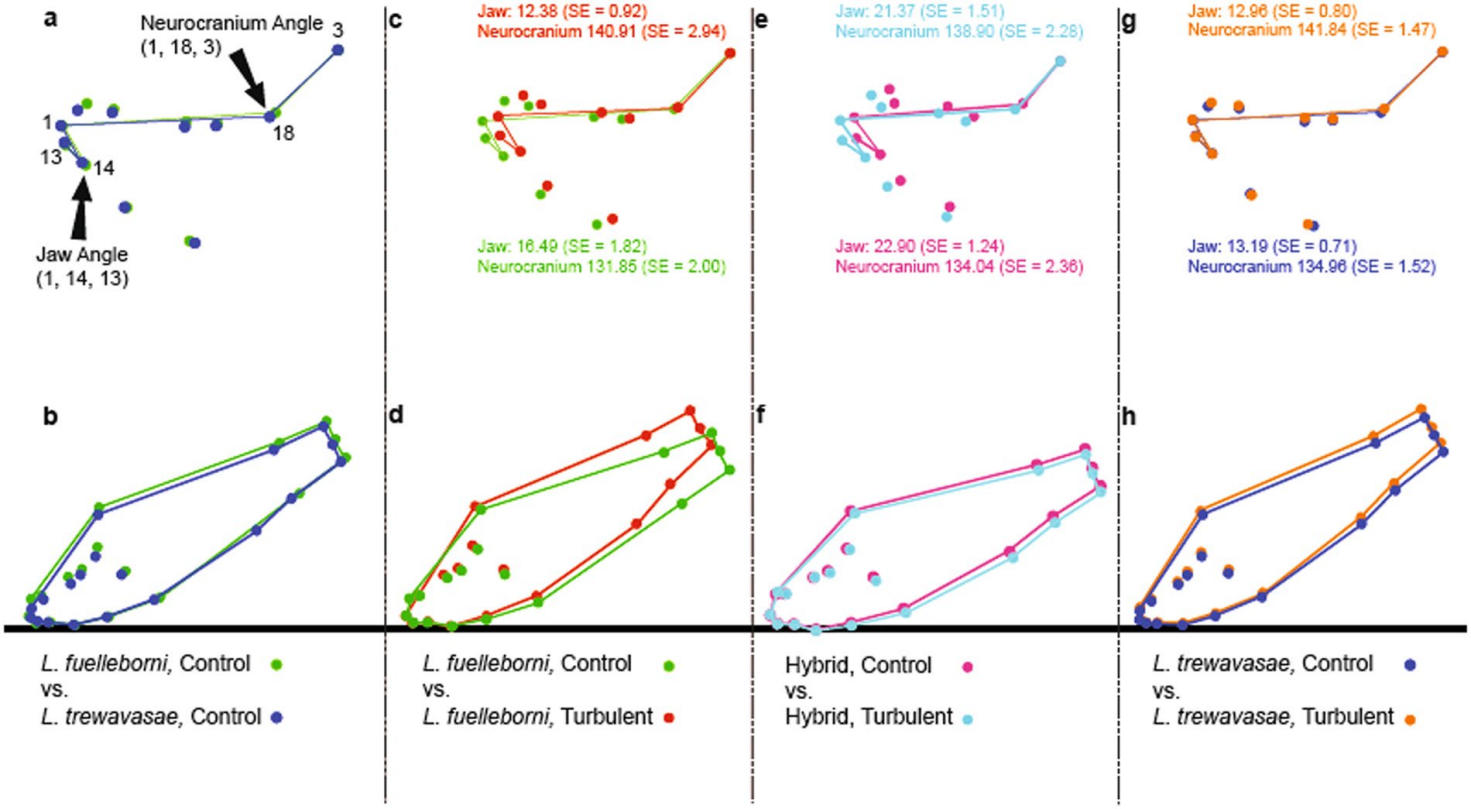
Comparisons of neurocranium and jaw angles among treatment groups, and examinations of possible feeding angles resulting from these morphological angles. Colors identify treatment groups, and are the same as in Fig. 2. From left to right: Panel (a) compares the jaw and neurocranium angles of *L*. *fuelleborni* control (green) and *L*. *trewavasae* control (blue), and further illustrates the landmarks that comprise each angle (jaw angle: landmarks 1, 14, 13; neurocranium angle: landmarks 1, 18, 3; see Fig. 5 in the Methods for a description of all landmarks). Panel (b) superimposes all landmarks of the control groups of *L*. *fuelleborni* and *L*. *trewavasae* as a heuristic to compare an approximate feeding position these fishes would have assumed in this experiment. Panel (c) comparison of jaw and neurocranium angles of *L*. *fuelleborni* control (green) and *L*. *fuelleborni* turbulent (red), including the average angle and standard error (SE) for both groups; Panel (d) superimposition of all landmarks of *L*. *fuelleborni* control and *L*. *fuelleborni* turbulent specimens. Panel (e) comparison of angles of hybrid control (pink) and hybrid turbulent (aqua); Panel (f) superimposition of all landmarks of hybrid control and hybrid turbulent. Panel (g) comparison of angles of *L*. *trewavasae* control (blue) and *L*. *trewavasae* turbulent (orange); Panel (h) superimposition of all landmarks of *L*. *trewavasae* control and *L*. *trewavasae* turbulent. Note the increased feeding angles in turbulent-raised *L*. *fuelleborni* and *L*. *trewavasae*, especially in comparison to their control-raised counterparts. See Table 2 for analyses of differences of angles among treatment groups.

Surprisingly, body depth did not differ among treatments; *L*. *trewavasae*, *L*. *fuelleborni*, and their hybrids did not adopt a deeper body in the turbulent treatment as opposed to the control. Instead, the primary differences between the turbulent and control fish in both the parental species and the hybrids are an upwards-tilted neurocranium and a more prominent snout (Fig. 2). The tilt of the neurocranium can be measured by comparing the angle formed by landmarks 1, 18, and 3 (the “neurocranium angle”); as well as the angle formed by landmarks 1, 14, and 13 (the “jaw angle”); among treatment groups. For all species, the neurocranium angle is greater in the turbulent group than in the control fishes, while the jaw angle is smaller for turbulent individuals than those raised in the control condition (Table 2; Fig. 3). Together, these two bodily angles conspire to increase the presumed feeding angle of turbulent-reared individuals, regardless of species (Fig. 3).

**Table 2:** Differences in jaw and neurocranium angles among species and treatments.

Jaw angle (n = 202; Multiple R^2^ = 0.302)					
Variable	Sum of Squares	df	Mean Square	F	p
Species	3056.101	2	1528.050	38.650	≤0.001
Treatment	172.633	1	172.633	4.367	0.038
Species*Treatment	117.215	2	58.608	1.482	0.230
Error	7748.972	196	39.536		
**Neurocranium angle (n** **=** **202; Multiple R**^**2**^ **=** **0**.**098)**					
Variable	Sum of Squares	df	Mean Square	F	p
Species	195.147	2	97.573	0.775	0.462
Treatment	2156.504	1	2156.504	17.132	≤0.001
Species*Treatment	109.292	2	54.646	0.434	0.648
Error	24671.545	196	152.875

### Selection

**Figure 4:**
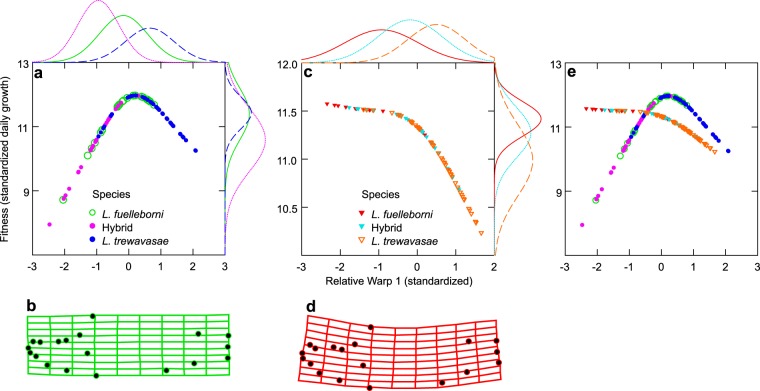
Fitness curves of control- and turbulent-raised specimens. Panel (a) depicts the fitness curve for body shape in the control condition; Panel (b) is the deformation grid that depicts the morphology with the highest fitness in this control treatment, which is a straight, somewhat deep body with no curvature to the neurocranium, similar to that found in *L*. *fuelleborni*. Panel (c) displays the fitness curve for the turbulent condition, and Panel (d) is a deformation grid depicting the morphology of maximum fitness depicted in the turbulent condition, which consists of a deep, somewhat curved body that results in an upturned neurocranium, which was found in *L*. *fuelleborni* and to a lesser degree in hybrids. The probability curves on the top and right sides of panels (a) and (c) show the distributions of RW1 scores and fitness, respectively. Panel (e) is a superimposition of the fitness curves, clearly displaying the directional selection against slender, straight bodies in the turbulent condition.

In the control condition, both *L*. *fuelleborni* and *L*. *trewavasae*, despite their radically different body shapes, have higher levels of fitness compared to the hybrids raised in this condition (Fig. 4a; Table 3). Maximal fitness in the control condition is primarily associated with a straight body, without any exaggerated curvature to the neurocranium (see deformation grid in Fig. 4b). Alternatively, by allowing young *Labeotropheus* to grow in the turbulent condition, there is a directional shift in the morphology that maximizes fitness (Fig. 4c). In the turbulent treatment, standardized daily growth is increased when the fishes adopt a morphology incorporating the upward-tilted neurocranium described above (see deformation in Fig. 4d). *Labeotropheus fuelleborni* raised under these conditions have the highest fitness, and *L*. *trewavasae* the lowest; interestingly, the hybrid *Labeotropheus* have a fitness intermediate to that of the parental species (Fig. 4c; Table 3). Further, when comparisons are made within species between treatments, there is no difference between treatments for *L*. *fuelleborni*, but both *L*. *trewavasae* (decrease in fitness from control to turbulent) and the *Labeotropheus* hybrids (increase in fitness from control to turbulent) do exhibit statistically significant differences in fitness (Table 3). This directional selection is made apparent when the fitness curves are superimposed on the same set of axes (Fig. 4e).

**Table 3:** Differences in fitness (Ŷ) among species within both treatments.

Control (n = 105; Multiple R^2^ = 0.212)					
Variable	Sum of Squares	df	Mean Square	F	p
Species	15.050	2	7.525	13.733	≤0.001
Error	55.894	102	0.548		
**Bonferroni-corrected pairwise differences in Ŷ among species in the Control treatment**					
		***L***. ***fuelleborni***		***Labeotropheus*** **hybrid**	
*Labeotropheus* hybrid		−0.804***			
*L*. *trewavasae*		0.037		0.841***	
**Turbulent (n** **=** **97; Multiple R**^**2**^ **=** **0**.**262)**					
**Variable**	**Sum of Squares**	**df**	**Mean Square**	**F**	**p**
Species	3.283	2	1.642	16.710	≤0.001
Error	9.235	94	0.098		
**Bonferroni-corrected pairwise differences in Ŷ among species in the Turbulent treatment**					
		***L***. ***fuelleborni***		***Labeotropheus*** **hybrid**	
*Labeotropheus* hybrid		−0.143			
*L*. *trewavasae*		−0.430***		−0.288***	

*p ≤ 0.05; **p ≤ 0.01; ***p ≤ 0.001.

## Discussion

The results of this experiment support the hypothesis that *L*. *trewavasae* would suffer a loss of fitness if raised in a turbulent environment; young *L*. *trewavasae* have a lower growth rate when grown in turbulent conditions than when raised in calm conditions. This reduced growth and concomitant loss of fitness is largely due to the fact that *L*. *trewavasae* failed to effectively adapt its morphology to the turbulent treatment. Conversely, the fitness of *L*. *fuelleborni* was equally high in both the turbulent and control conditions, largely due to the adoption of an upturned neurocranium and enlarged snout when raised in turbulence. The *Labeotropheus* hybrids presented a more complicated situation. Surprisingly, they developed an upturned neurocranium and enlarged snout in the control treatment, which was not advantageous under these conditions, resulting in the lowest fitness of any of the species. In the turbulent treatment, their fitness increased significantly due to the adoption of a craniofacial morphology similar to that of *L*. *fuelleborni* under these conditions. Overall, exposure to turbulence resulted in directional selection against a slender body and straight or downturned head, instead favoring deeper bodies with an upturned neurocranium.

Although not directly measured in these experiments, the upturned neurocranium would actually increase the typical feeding angle of the fish^13,17^. As such, instead of feeding more or less parallel to the surface, gleaning algae as they swim, the turbulent-reared purebred fish, especially *L*. *fuelleborni*, would be feeding in a somewhat exaggerated “headstand,” similar to the feeding angles employed by species of *Maylandia*^13,17^. In this position, the prominent snout pad would likely be firmly pressed against the feeding surface while the fish swims forward, using the snout pad not as a fulcrum, but as a stabilizer, thus allowing it to maintain its position while feeding in the turbulence. While feeding angles among Lake Malaŵi cichlids vary depending upon the orientation of the substrate^13^, the feeding surface in this experiment was constant and consistent between treatments, strongly suggesting that turbulence was responsible for the changes to the neurocranium.

Given *L*. *trewavasae*’s preference for deeper habitats, where they are less likely to encounter wave-generated turbulence, it is not necessarily surprising that they struggled to grow under laboratory conditions that mimicked such turbulence. Additionally, although it was not tested in this experiment, the body shape typical of *L*. *trewavasae* is hypothesized to be an adaptation for feeding on the vertical surfaces of rocks, or within the crevices among rocks^3,9^. As such, forcing this species to feed on a horizontal surface in turbulence could represent a significant energetic challenge that could account for its impaired growth. Recent laboratory experiments demonstrate that, in conditions without turbulence, *L*. *trewavasae* both executes more bites and beats its fins more frequently when feeding on horizontal surfaces^13^, thus likely expending more energy. Conversely, it is not surprising that *L*. *fuelleborni* performed well and had high fitness under both conditions, given that these deeper bodied fish were not challenged to feed in an environment that might have been more favorable to *L*. *trewavasae*.

The differences in hybrid fitness and morphology between the treatments are puzzling. In the control condition, the hybrids had an upwardly-tilted and expanded neurocranium, perhaps extremely so. This morphology is not found in either of the parental species raised in this condition; indeed, it is very different from that of either parental species’ control cohorts, which might explain the poor performance of the hybrids in this condition. As such, this appears to be a transgressive phenotype^18,19^, though one that is poorly matched to this environment. On the other hand, in the turbulent treatment, the hybrids adopt a morphology very similar to that of the parental species, which better met the demands of the turbulent condition, and likely explains the increased fitness of the hybrids raised in this environment.

The deeper bodies found in the fittest individuals in this experiment likely made them more massive, giving them more inertia with which they could resist turbulence, thus allowing them to forage more efficiently in the turbulent treatment sensu^3^. Not coincidentally, this mimics the pattern found in *Labeotropheus* in Lake Malaŵi; deep-bodied *Labeotropheus* are found in wave-swept nearshore areas, while their slender congeners are found in deeper waters. This relationship between body depth and depth distribution becomes somewhat puzzling when considering the depths at which other rock-dwelling cichlids are found in Lake Malaŵi. Many other genera of these fishes, including *Maylandia*, *Tropheops*, and *Petrotilapia*, among others, are always deep-bodied, no matter the depths at which they live. Interestingly, the species within these genera employ much more elevated feeding angles than do the *Labeotropheus*; based upon *in situ* observations, *Maylandia*, *Tropheops*, and *Petrotilapia* achieve feeding angles of 58°–90°, while the *Labeotropheus* have the lowest feeding angle of all of the rock-dwelling cichlids of Lake Malaŵi, ranging between 35° and 45°^17^. As we hypothesize above, these elevated feeding angles likely offer greater stability to these species, and would be useful in any habitat, but especially in turbulent areas. The *Labeotropheus*, on the other hand, are restricted to much more acute feeding angles by their characteristic snout; as such, the only way for them to achieve higher angles, and the greater stability offered by such positions, would be via an upturned neurocranium.

Given that the parental specimens used to generate the experimental fishes were obtained from the same location in Lake Malaŵi, these results also provide insight on the likelihood of both the appearance and survival of hybrid *Labeotropheus* in the wild. Any hybridization between *L*. *fuelleborni* (or other deeper-bodied *Labeotropheus*) and *L*. *trewavasae* (or other slender *Labeotropheus*) would require one or the other parental species to at least temporarily exploit a habitat for which it would be poorly adapted. In the case of *L*. *fuelleborni*, it would have to swim to a depth below which it is commonly found (≥8 m^3,9^), and to which it cannot acclimate^3^, while *L*. *trewavasae* would have to negotiate the turbulent environments in which it would have difficulty feeding and maintaining mass (this study), as well as having to compete with more aggressive and strongly territorial male *L*. *fuelleborni*^3,9^. The hybrids used in this experiment, while viable and fertile (an inbred F_2_ generation currently exists in our laboratory aquaria; Pauers, unpubl. data), are not likely to exist in Lake Malaŵi. While they could likely thrive in a shallow, turbulent environment, their paternal species, *L*. *trewavasae*, could not. Similarly, although it might be possible for their maternal species, *L*. *fuelleborni*, to visit a deeper depth, perhaps at an island or submerged reef^3,9^, the hybrid young could not effectively compete with either parental species in these deep, calm habitats.

Interestingly, our results suggest that both selection on morphology and hybridization in the *Labeotropheus* would have been concomitant with water level fluctuations in Lake Malaŵi. Throughout its history, Lake Malaŵi has undergone extreme lake level fluctuations, ranging from its current deep, full-basin level, to nearly dry about 100,000 years ago^20,21^. A decrease in lake level would have severely limited the rock-strewn habitats favored by the *Labeotropheus*, especially the deeper rocky shelves preferred by *L*. *trewavasae*^20,21^. As such, the available rocky habitat would have been shallow and likely turbulent, thus favoring a morphology that could successfully feed under these conditions; i.e., a deeper body and an upturned neurocranium. Further, any deep-dwelling, slender *Labeotropheus* would have likely been forced to hybridize with the deeper-bodied *Labeotropheus* sensu^21^; interestingly, the predominant shallow, turbulent conditions surrounding these rocky habitats would have been ideal for *Labeotropheus* hybrids, given the morphology we found them to adopt under these conditions. Once lake levels rose, and the deep rocky habitats reappeared, selection against slender *Labeotropheus* morphotypes would relax, and individuals with this body shape could once again exploit these habitats. Conversely, selection against hybridization would strengthen, due to assortative mating based on male nuptial coloration^22^, and would help to promote the subsequent radiation of *Labeotropheus*^21^.

Directional selection has been a potent force in the evolution of the cichlids of Lake Malaŵi, and has been highly influential in the evolution of the oral jaws of *L*. *fuelleborni*^23^; as such, it is not surprising to find that it is also active in shaping the morphology of the neurocranium, snout, and body, as demonstrated in our current experiments. Feeding in turbulence imposes finite constraints on both the morphology and swimming performance of fishes reviewed in^24^, thus establishing a selective environment favoring what are often extreme morphologies^24,25^. The experiments described herein placed fish that already have one of the most extreme morphologies of any cichlid from Lake Malaŵi^8^ in an experimental condition in which the morphology had to be further elaborated in order for these fishes to grow. The stocky, robust, *L*. *fuelleborni*, ostensibly specialized for feeding in turbulence, readily adapted to the experimental turbulence. On the other hand, the slender *L*. *trewavasae*, long suggested to be specialized for feeding in calm, structurally-complex habitats, was unable to effectively feed and grow in the experimental turbulent condition. It seems likely, then, that directional selection is responsible for *L*. *fuelleborni*’s successful exploitation of the shallow, turbulent nearshore regions of Lake Malaŵi.

## Methods

### Experimental animals

Wild caught adult specimens of *Labeotropheus fuelleborni* and *L*. *trewavasae* were obtained from a reputable Malaŵi-based exporter of cichlid fishes (Stuart M. Grant, Ltd.); both species were captured at the Chidunga Rocks in southwestern Lake Malaŵi. Twenty individuals of each species were obtained, ten of each sex. These adults were segregated by species and sex into four separate 160 L aquaria and were housed at the Aquarium and Reptile Center of the Milwaukee County Zoo. These fishes were fed to satiation daily with a mixture of spirulina flake food (e.g., Formula Two; Ocean Nutrition, Newark, CA, USA), cichlid pellets (e.g., Hikari Cichlid Excel; Kyorin Co., Ltd., Hyogo, Japan), and a variety of frozen foods (e.g., bloodworms, brine shrimp; various manufacturers). Water temperature was maintained at 28 °C, and water quality was maintained using standard box filters and twice-weekly 50% water changes.

In order to produce the experimental fishes, haphazardly-selected groups of adults (1–3 males with 2–5 females) were moved into separate 160 L aquaria, which were maintained as described above. Brooding females were isolated until their fry were released at about 21 days post-fertilization. Hybrids were produced by isolating male *L*. *trewavasae* with female *L*. *fuelleborni*. The sample sizes of individuals ultimately harvested from each replicate are shown in Table 4.

**Table 4:** Sample sizes of individuals raised 45 days in each treatment and replicate.

Species	Treatment	Replicate	n
*L*. *fuelleborni*	Control	1	17
		2	6
	Turbulent	1	17
		2	5
Hybrid	Control	1	26
		2	5
	Turbulent	1	17
		2	8
*L*. *trewavasae*	Control	1	22
		2	29
	Turbulent	1	20
		2	30

### Experimental rearing conditions

When the fry were between 25 and 35 days post fertilization, they were then moved to the experimental mesocosms. Two such mesocosms were built using large, 1.83 m diameter aquaculture tubs. The tubs were filled to a depth of 56 cm, giving a wetted volume of 1470 L, and were heated to a temperature of 28 °C using programmable titanium heaters. Filtration was provided by single submersible filter (Fluval U4; Rolf C. Hagen Corp., Mansfield, MA, USA) that produced a current of 260 L*h^−1^. The wastes that accumulated on the bottom of each mesocosm were regularly removed via siphoning, and approximately 10% of the volume of each mesocosm was removed and replaced each week.

A stack of paving blocks 53.33 cm L  ×  30.5 cm W  ×  40.6 cm H was used as a feeding station. Feeding blocks were created by smearing a gel-based vegetarian food (Mazuri Herbivore Aquatic Gel Diet – No Corn; PMI Nutrition, Inc., St. Louis, MO, USA) onto the upper surface (15.25 cm × 15.25 cm; 232.56 cm^2^) of a paving block, which was then air-dried overnight and stored in a refrigerator at about 8 °C. These feeding blocks were placed atop the feeding station and replaced when all the food had been eaten. The feeding blocks were an important part of this experiment, as they allowed the young *Labeotropheus* to feed naturally (i.e., by scraping food from a surface), and forced them to forage in the experimental turbulence (see below).

The control mesocosm was set up exactly as described above, and had a background current of approximately 0.017 m*s^−1^. The turbulent mesocosm had two additional submersible water pumps mounted directly across from the feeding tower, with their outputs aimed at the feeding block. These pumps (Marineland MaxiJet Pro 1200; Spectrum Brands, Blacksburg, VA, USA) were each capable of producing a current of 4920 L*h^−1^, but were powered by a wave generator (Ocean Pulse Quadra; Transworld Aquatic Ent,; Inglewood, CA, USA) that created three pulses of water every minute (i.e., one pulse every 20 s). Each wave travelled at a rate of 0.12 m*s^−1^, producing the turbulent conditions in which these fishes were forced to feed.

The young fish were raised in each condition for 45 days, then removed and euthanized with an overdose of MS-222 (tricaine methanesulfonate; 250 mg*L^−1^). The fish were then stored in 70% EtOH until they could be photographed for morphometric analysis.

All of the procedures described above were approved by the UW Colleges Animal Care and Use Committee (ACUC), protocol # 2017-MP-03R, and were also approved by the Senior Animal Staff of the Milwaukee County Zoo (MCZ). All procedures were performed in accordance with the relevant guidelines and regulations set forth by the UW Colleges ACUC and the MCZ Senior Animal Staff, and followed all legal requirements of the United States of America.

### Geometric morphometric analysis

**Figure 5:**
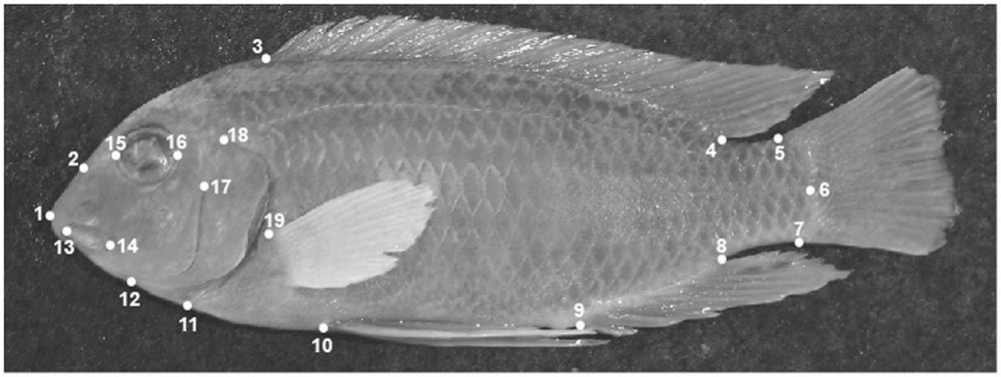
Homologous landmarks used for geometric morphometric analysis. For clarity, the landmarks are displayed on an adult *L*. *fuelleborni* from Chidunga Rocks. The landmark positions are described in greater detail in the Methods.

Preserved fishes were pinned with fins erect onto a felt-covered Styrofoam sheet and photographed from a distance of 10 cm with a digital camera attached to a copystand. These photographs were then imported to ImageJ and 19 homologous landmarks were superimposed onto the images. These landmarks, following^26^, were: (1) anterior tip of snout, (2) dorsal tip of premaxillary pedicel, (3) origin of dorsal fin, (4) insertion of dorsal fin, (5) dorsal insertion of caudal fin, (6) caudal border of hypural plate aligned with lower lateral line, (7) ventral insertion of caudal fin, (8) insertion of anal fin, (9) origin of anal fin, (10) base of pelvic fin spine, (11) a point on posterior margin of opercular membrane meeting ventral margin of head, (12) posterior end of dentary symphysis, (13) anteriormost margin of upper jaw soft tissue, (14) posterior end of jaws, (15) anterior margin of midline through the eye, (16) posterior margin of midline through the eye, (17) dorsal end of preopercle, (18) dorsalmost end of opercule, and (19) origin of pectoral fin (Fig. 5).

The (x, y) coordinates of each landmark for each specimen were then used in a Generalized Procrustes Analysis^27^ in the program Coordgen8 to reduce the effects of size and orientation of each specimen. Next, a multiple regression of shape on geometric centroid size was performed using the Standard6 software to remove the effects of allometry from the data. Allometry-free data were then subjected to a thin-plate spline procedure to generate geometric descriptors of shape variation called relative warps (RW); this procedure was performed using the program TPSRelW. All deformation grids were generated with TPSRelW. Additionally, the angles among landmarks were calculated using the TradMorphGen tools within Coordgen8. All morphometric software is free for public use and can be downloaded from: http://www.life.bio.sunysb.edu/morph/. Additionally, we used Systat 10.0 to calculate the ANOVAs of the RW scores, and to create the bivariate plots of the RW scores.

### Selection analyses

In order to estimate the possible action of selection on morphology, we first estimated the daily growth rate for each individual over the course of the 45-day experiment by subtracting the mean standard length (SL) of a cohort of 10 individuals of the same species collected and euthanized at day 0 of the experiment from its SL at day 45. Since RW1 explains more variation than any of the other RW factors, and because it describes morphological variation among both species and treatments, we used that as our proxy of body shape. We then standardized each individual’s RW1 score by subtracting the mean RW1 score of its experimental group from its own RW1 score, and dividing by the standard deviation of the group’s RW1 scores^28^. These data were then used to estimate fitness with a cubic spline in the program glmsWIN 1.0^29^, which can be downloaded from: https://www.zoology.ubc.ca/∼schluter/wordpress/software/.

## Data Availability

The datasets generated during and/or analyzed during the current study are available from the corresponding author on reasonable request.
